# Hemorrhagic Ascites in Chronic Lymphocytic Leukemia With Immunoglobulin Heavy Chain Variable Region 3-21 (IGHV3-21) Usage: A Case Requiring Individualized Treatment

**DOI:** 10.7759/cureus.106515

**Published:** 2026-04-06

**Authors:** Suhail Sapkota, Niket Shah, Maha Bayya, Jasneet Gill, Shreebridhi Pande, Shreya Motkur, Sai Sushrutha Mudupula Vemula, Borys Hrinczenko

**Affiliations:** 1 Internal Medicine, University of Michigan Health-Sparrow, Lansing, USA; 2 Hematology and Oncology, Karmanos Cancer Institute at McLaren Greater Lansing, Lansing, USA; 3 Internal Medicine, North Knoxville Medical Center, Powell, USA; 4 Internal Medicine, Cape Fear Valley Health, Fayetteville, USA

**Keywords:** chronic lymphocytic leukemia, hemorrhagic ascites, ighv3-21, iwcll, prognostic markers

## Abstract

Chronic lymphocytic leukemia (CLL) typically follows an indolent course, with therapy initiated only when patients meet the International Workshop on CLL (iwCLL) treatment criteria. Immunoglobulin heavy chain variable region (IGHV) mutation status is an established prognostic marker, with mutated IGHV associated with delayed time to first treatment and more favorable outcomes. However, IGHV3-21 gene usage introduces prognostic complexity, especially in the absence of subset-level immunogenetic analysis. We report the case of a man in his 50s with stable, mutated IGHV CLL who presented with hemorrhagic ascites, a rare and atypical complication. Ascitic fluid analysis demonstrated grossly bloody, lymphocyte-predominant fluid with flow cytometric evidence of clonal CLL involvement. Imaging showed no bulky lymphadenopathy, splenomegaly, cirrhosis, or portal hypertensive features. Despite molecular features associated with a favorable prognosis, the severity of his presentation prompted the initiation of therapy after the exclusion of alternative etiologies. This case highlights the diagnostic and therapeutic uncertainty created by atypical symptomatic presentation of CLL and the prognostic ambiguity of IGHV3-21 CLL requiring cautious interpretation.

## Introduction

Chronic lymphocytic leukemia (CLL) usually follows an indolent course, and treatment is typically reserved for patients who meet the International Workshop on CLL (iwCLL) criteria for active disease [1,2]. Among prognostic biomarkers, immunoglobulin heavy chain variable region (IGHV) mutation status has emerged as a key predictor of clinical outcomes, with mutated IGHV associated with prolonged survival and delayed time to first treatment [3]. However, the adverse prognosis historically attributed to IGHV3-21 usage is primarily driven by a stereotyped subset (IGHV3-21/immunoglobulin lambda variable 3-21 (IGLV3-21)) rather than being uniform across all IGHV3-21-utilizing cases [4]. The clinical significance of IGHV3-21/IGLV3-21 must be weighed alongside other factors such as TP53 status, cytogenetic profile, complex karyotype, β2-microglobulin, and disease burden. Ascites is uncommon in CLL, and hemorrhagic ascites is an even more unusual presentation. While current frontline therapy for CLL favors Bruton tyrosine kinase (BTK) or B-cell lymphoma 2 (BCL-2) inhibitors, this case illustrates an atypical complication with diagnostic challenge leading to treatment initiation.

## Case presentation

A man in his early 50s with a 3.5-year history of CLL under active surveillance presented with several months of progressive abdominal distension, bloating, and early satiety with worsening abdominal pain during the week before presentation. The patient had no relevant social or family history. He denied fever, weight loss, night sweats, or new lymphadenopathy. Physical examination revealed shifting dullness consistent with ascites but no peripheral edema or hepatosplenomegaly.

**Figure 1:**
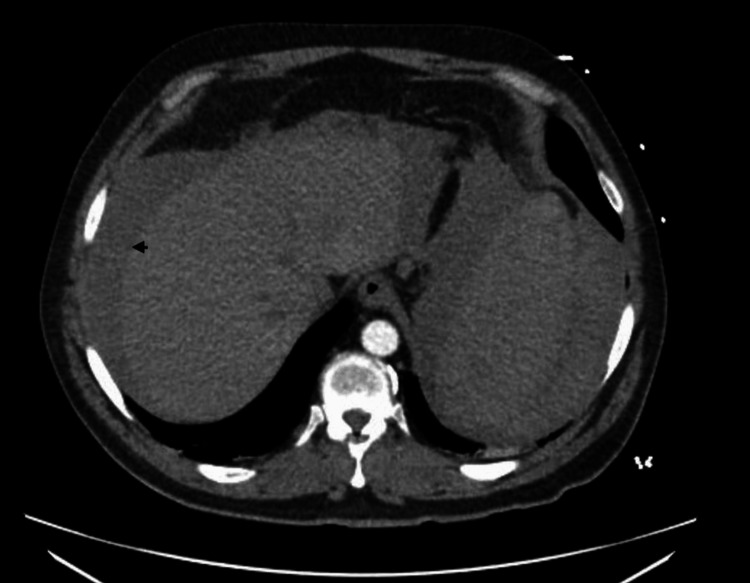
Computed tomography of the abdomen with arrow indicating moderate-to-large ascites. No lymphadenopathy, splenomegaly, cirrhosis, or features of portal hypertension were identified.

Abdominal ultrasound confirmed moderate-to-large ascites. Diagnostic and therapeutic paracentesis was performed and yielded 1 L of cloudy, dark red, grossly bloody fluid. Fluid analysis showed a red blood cell (RBC) count of 2,288,303/µL, a total nucleated cell count of 28,920/µL, 92% lymphocytes, 3% neutrophils, 4% monocytes/macrophages, and 1% eosinophils. Abdominal fluid protein was 4.3 g/dL, glucose 76 mg/dL, and lactate dehydrogenase (LDH) 308 U/L. Ascitic albumin was 3.2 g/dL, yielding a serum-ascites albumin gradient (SAAG) of 0.7 g/dL, consistent with a non-portal hypertensive process. Ascitic fluid culture showed no growth. Computed tomography imaging demonstrated no lymphadenopathy, splenomegaly, or signs of cirrhosis (Figure 1).

**Figure 2:**
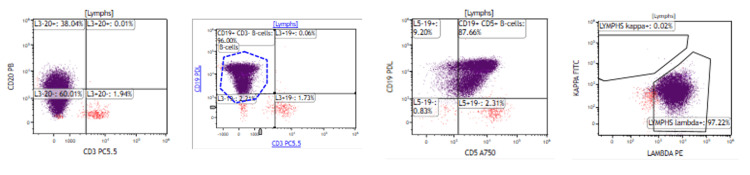
Flow cytometry of ascitic fluid showing clonal B cells positive for CD19, CD5, dim CD20, and kappa light chain restriction, consistent with CLL. CD19: cluster of differentiation 19; CD5: cluster of differentiation 5; CD20: cluster of differentiation 20; CLL: chronic lymphocytic leukemia

Flow cytometry of the ascitic fluid revealed a monoclonal B-cell population consistent with the patient's known CLL (Figure 2).

Laboratory evaluation revealed a white blood cell count of 57.3×10⁹/L, with an absolute lymphocyte count of 51.57×10⁹/L, a hemoglobin of 10.1 g/dL, a platelet count of 102×10⁹/L, and a normal renal and hepatic function. Serum albumin was 3.9 g/dL, serum LDH was 110 U/L, and total serum protein was 5.2 g/dL. Molecular testing demonstrated a mutated IGHV with IGHV3-21 usage and wild-type TP53. Peripheral blood flow cytometry demonstrated a cluster of differentiation 5 (CD5)-positive B-cell population consistent with CLL, and fluorescence in situ hybridization (FISH) showed isolated heterozygous deletion of 13q14.3, with no del(11q), del(17p), or trisomy 12. Expanded next-generation sequencing panel testing and zeta-chain-associated protein kinase 70 (ZAP-70) analysis were not performed as part of the clinical workup.

The differential diagnosis for hemorrhagic ascites included peritoneal carcinomatosis, which was ruled out by cytology and imaging; portal hypertension, which was excluded based on imaging and normal liver function tests; tuberculous peritonitis, for which there was no clinical or laboratory evidence; and peritoneal involvement by CLL, which was confirmed by flow cytometry. The low SAAG further supported a non-portal hypertensive etiology. Taken together, the grossly bloody lymphocyte-predominant ascites, negative culture, absence of malignant epithelial cells, and presence of clonal CLL cells in the fluid made leukemic peritoneal involvement the most likely explanation, although a definitive mechanism for hemorrhage could not be established.

After multidisciplinary discussions at multiple academic centers, the patient elected to initiate fludarabine, cyclophosphamide, and rituximab therapy, citing long-term remission data in younger, fit patients with mutated IGHV. Although current frontline treatment generally favors targeted therapy, this decision followed discussion of risks, benefits, and available alternatives, including fixed-duration targeted approaches. Pretreatment neutropenia was managed with granulocyte colony-stimulating factor support using filgrastim. Although fludarabine, cyclophosphamide, and rituximab is no longer recommended as standard first-line therapy, it was selected after multidisciplinary input and patient preference for a time-limited treatment approach.

The patient tolerated the therapy well, with the resolution of ascites and improvement in abdominal symptoms after two cycles of treatment. At the short-term follow-up, only a single paracentesis was required prior to the initiation of treatment. While the initial response at one month post-treatment is encouraging, longer-term follow-up is required to assess the durability of remission with this regimen and its long-term safety profile in the setting of an atypical presentation.

## Discussion

This case illustrates the nuanced prognostic implications of IGHV3-21 usage in CLL. While mutated IGHV is generally associated with favorable outcomes, IGHV3-21 cases, particularly those belonging to subset number 2, may demonstrate a more aggressive behavior [4,5]. Subset-level immunogenetic analysis, however, is not widely available in routine clinical practice, creating uncertainty in risk stratification for patients with IGHV3-21 usage [5,6]. More broadly, contemporary CLL risk assessment integrates IGHV status with additional clinical and biologic factors, including cytogenetic abnormalities, TP53 status, and overall disease burden, rather than relying on a single immunogenetic feature in isolation.

Hemorrhagic ascites as a presenting complication of CLL is rarely described in the literature and remains an unusual manifestation in routine clinical practice [7,8]. Potential mechanisms include direct peritoneal infiltration of leukemic cells, increased vascular permeability, or portal hypertension from leukemic infiltration [6]. In the present case, the presence of clonal CLL cells in ascitic fluid supports leukemic involvement of the peritoneal compartment, but does not independently establish causality for hemorrhage. However, the combination of grossly bloody lymphocyte-predominant ascites, negative cultures, absence of malignant epithelial cells, lack of cirrhosis or portal hypertensive features on imaging, and an SAAG of 0.7 g/dL supporting a non-portal hypertensive cause makes a CLL-related mechanism the most plausible explanation.

In this patient, hemorrhagic ascites represented a symptomatic and uncommon complication that prompted treatment initiation despite the absence of bulky progressive lymphadenopathy, symptomatic splenomegaly, or constitutional symptoms typically emphasized in standard iwCLL indications. The decision to pursue fludarabine, cyclophosphamide, and rituximab therapy was based on patient age, performance status, disease characteristics, and informed patient preference following multidisciplinary consultation. This rarity, combined with the prognostic uncertainty associated with IGHV3-21, made management particularly challenging and highlights a gap in evidence-based guidance for atypical disease presentations.

Contemporary randomized trials have demonstrated superior progression-free survival with combinations of BTK and BCL-2 inhibitors compared with chemoimmunotherapy in treatment-naïve CLL, establishing targeted therapy as a frontline standard [9]. However, long-term remissions with fludarabine, cyclophosphamide, and rituximab remain well documented in younger, fit patients with mutated IGHV and no high-risk cytogenetics [10]. Fludarabine, cyclophosphamide, and rituximab has historically been associated with deep and durable remissions in selected younger, fit patients with mutated IGHV and favorable cytogenetics. In this case, the patient's age, fitness, mutated IGHV status, absence of TP53 disruption, and preference for a time-limited approach informed the treatment decision after multidisciplinary discussion. This choice should not be interpreted as an endorsement of chemoimmunotherapy as standard frontline treatment. Given the known risks of this regimen, including prolonged cytopenias, infectious complications, and secondary malignancies, longer follow-up is necessary to assess the durability of response and long-term safety [10].

This case underscores the importance of individualized clinical judgment in CLL management and illustrates how atypical manifestations may justify deviation from conventional treatment algorithms.

## Conclusions

Hemorrhagic ascites is a rare but recognized complication of CLL and may prompt treatment consideration in selected patients after the careful exclusion of alternative etiologies. It is important to have individualized clinical judgment when a patient presents with atypical symptomatic manifestations that fall outside conventional iwCLL treatment standards and should not be interpreted as redefining treatment thresholds. IGHV3-21 gene usage in CLL introduces prognostic complexity, particularly when subset-level immunogenetic data are unavailable. Individualized management is essential in CLL when atypical or aggressive clinical features fall outside standard risk stratification models. This report does not advocate the routine use of fludarabine, cyclophosphamide, and rituximab in current practice but highlights that treatment decisions may be individualized based on patient characteristics, disease features, and informed patient preference rather than standard indicators alone. 
